# Comparing Nutrient Profiles of Meat and Fish with Plant-Based Alternatives: Analysis of Nutrients, Ingredients, and Fortification Patterns

**DOI:** 10.3390/nu16162725

**Published:** 2024-08-16

**Authors:** Adam Drewnowski, Maaike J. Bruins, Julia J. F. Besselink

**Affiliations:** 1 Center for Public Health Nutrition, University of Washington, Box 353410, Seattle, WA 98195, USA; 2 dsm-firmenich, Taste, Texture and Health, 2613 AX Delft, The Netherlands; maaike.bruins@dsm-firmenich.com (M.J.B.); julia.besselink@dsm-firmenich.com (J.J.F.B.)

**Keywords:** plant-based products, plant protein, meat and fish alternatives, vegan, nutrient composition, nutrient profiling, ingredient list, fortification, USDA Food Database

## Abstract

Nutrient quality of plant-based meat and fish alternative (MFA) products has been reported as variable. The present objective was to compare the nutrient density of raw meat and fish with MFA products and to examine MFA ingredients and fortification patterns. Nutrient composition data for raw beef, pork, and chicken (*n* = 450) came from the USDA Standard Reference Legacy Reselase (SR28) nutrient composition database (*n* = 450). Data for plant-based meat alternatives (*n* = 118) came from the USDA Branded Food Products Database (BFPDB). Data for fish and seafood (*n* = 68) and alternatives (*n* = 12) came from the Danish Frida Database and Mintel (*n* = 503). Analyses compared macronutrient content and selected micronutrients per 100 g and percentage of US Daily Values. Nutrient density scores were based on the NRF5.3 algorithm. NRF5.3 scores were significantly lower for MFA products as compared to meat or fish. Saturated fat content was lower, but the mean MFA sodium content was 660 mg/100 g compared to 60 mg/100 g for beef. Mean protein content (17 g) was lower than for beef and pork (20 g). A search of ingredient lists found that fortification with most nutrients associated with animal source proteins (such as B vitamins, vitamin D, iodine, zinc, and omega-3 EPA and DHA) was minimal. Plant-based MFA products would benefit from nutrient standards and fortification guidelines to ensure that these products are not nutritionally inferior to the originals.

## 1. Introduction

The adoption of plant-based diets has been reported to improve diet quality [1], provide cardiometabolic benefits [2,3,4], improve animal welfare, and lower the impact of food production on the environment [2,5]. Plant-based meat and fish alternative (MFA) products are intended to substitute for beef, pork, chicken, fish, or seafood [6]. Offered in the form of meatless burgers, meatballs, sausages, nuggets, filets, or salmon and tuna substitutes, MFA products promise to deliver equivalent nutrition and maintain the satisfaction and the sensory qualities of the original food [7]. Plant-based alternatives are seen as key contributors to plant-forward diets and are becoming an important market segment, at least in high-income countries [8,9].

However, the nutrient quality of plant-based MFA has been reported as variable. Whether they provide equivalent nutritional value is not yet clear. The US Food and Drug Administration (FDA) has not acted on the labeling of MFA products, and no fortification or labeling standards for MFA products have been developed. No regulatory standards for MFA products have been proposed by EFSA at this time. Seeking to address this research gap, the present analyses examined nutrient density, fortification patterns, and key ingredients of MFA products available in the US and Europe.

This investigation is timely, given recent initiatives in the EU and elsewhere. Having reached peak meat consumption [10], high-income countries are looking for ways to lower the animal-to-plant protein ratio in the population diet. Both Canada [11] and the Netherlands [12] have incorporated environmental impact in their dietary guidelines. The Health Council of the Netherlands has advocated a diet with a 60:40 ratio of plant to animal proteins [13]. Twenty-five city governments globally have supported the 2021 Plant-Based Treaty to create a “plant-based capital”, thereby stemming the impact of climate change [14]. The influential EAT-Lancet report has proposed a single planetary health diet with most of the dietary energy and the bulk of the protein coming from plants [15]. Beef and pork consumption in the EAT-Lancet planetary health diet was restricted to about 7 g each per person per day [16].

Novel plant protein-based foods are reputed to generally have a lower environmental footprint compared to most animal proteins [17]. Whether plant-based MFA products are also nutritionally equivalent to meat, poultry, and fish is not yet clear. Some reports suggest that the MFA products offer comparable, if not improved, overall nutritional value relative to meat [18,19]. However, other reports suggest the opposite [20,21]. The nutrient density of MFA products relative to animal-source foods needs to be evaluated using rigorous nutrient profiling methods and close examination of ingredient lists. Provided that MFA products are appropriately formulated and fortified with key nutrients, they potentially could be as nutritious as the products they attempt to simulate [22,23].

Food fortification can enhance the nutritional value of MFA products by adding nutrients that are present in the original animal-source foods [24,25]. For example, plant-based milk alternatives in the US are often fortified with calcium, vitamin A, and vitamin D, so their nutritional profile is more on par with milk [26]. The US FDA has now drafted nonbinding guidance to the industry that plant-based milk alternatives that use the term “milk” ought to declare on the product label how they are nutritionally different from cow milk [27]. The relevant nutrients where differences were found were protein, calcium, vitamin A, vitamin D, magnesium, phosphorus, potassium, riboflavin, and vitamin B12. The present analyses identified potential targets for fortification for MFA products.

## 2. Materials and Methods: Food Composition Databases

### 2.1. Meat and Poultry from SR28 Legacy Database (SR28)

Food composition data for raw meat and poultry came from the USDA National Nutrient Database for Standard Reference Legacy Release 28 (SR28), publicly available on FoodData Central [28]. The SR28 provides data on energy and nutrient content for 8789 foods derived from analysis, calculations, and/or scientific literature. Machine searches were performed for beef, pork, and chicken food items further identified as “raw”. Ground beef, ground pork, and ground chicken meat were included. Excluded were processed and cured meats and cooked meat products that were breaded, salted, fried, or deep fried, as well as meat-containing soups, stews, sandwiches, pizza, and other mixed foods. The commonly used version of SR28 does not contain data on iodine. The SR28 lists a very limited number of MFA products.

### 2.2. Plant-Based Alternatives from USDA Branded Food Products Database (BFPD)

Food composition data for packaged plant-based MFA came from the USDA BFPD, publicly available on FoodData Central [29]. Products were limited to selected categories: frozen dinners, entrees, appetizers, frozen patties and burgers, and other meats. The products had to have a meat-related term in the product name, such as burger, meatball, sausage, chicken, fish, shrimp, or crab. Machine searches of product names were used to identify products described as “meatless” or “vegan”. Ambiguous items such as “cheesy nuggets” or “veggie patties” were excluded, as were products that included eggs or dairy on the ingredient list, so the final list consisted of vegan products. Although very large (>400,000 items), the BFPD only contains those nutrients that are listed on the Nutrition Facts Panel (vitamins A, C, D, and calcium, iron, and potassium) and those for which a health claim is made. The BFPD also contains an electronic ingredient list, which was machine-searched for additives such as salt, sugar, protein concentrates and isolates, methylcellulose, and maltodextrin. Additional searches were performed to identify MFA products with added iron, zinc, calcium, magnesium, and selected vitamins and minerals that are commonly associated with meat, poultry, and fish.

### 2.3. Fish and Plant-Based Alternatives from Frida Food Database (Frida) and Mintel

Food composition data for fish, seafood, and plant-based foods came from the Frida Food Database Version 5.1 November 2023 (Denmark) [30]. The relevant food groups were lean fish, somewhat oily fish, oily fish, shellfish and products, and mollusks and their products. The Frida database had very limited information on plant-based products.

Additional data for plant-based seafood alternatives launched in North America and Europe came from Mintel GNPD. The selection was limited to products described as “fish”, “tuna” or “salmon” and launched between 1998–2024, with a majority launched after 2018. Relaunches or products with new packaging were excluded.

### 2.4. Nutrient Profiling of Meat, Poultry, Fish, and MFA Products

The NRF5.3 algorithm calculated per 100 g was used to assess the nutrient density of meats, fish, and MFA products. The positive NR5 sub-score was based on five nutrients to encourage: protein, iron, zinc, selenium, and vitamin B12. The negative LIM score was based on 3 nutrients to limit: saturated fat, total sugar, and sodium. NRF scores were calculated as the sum of percent daily values (%DV) for 5 nutrients to encourage minus the sum of %DV for the 3 nutrients to limit. All % DV were per 100 g and were capped at 100%. The final NRF5.3 score is given by: NRF5.3 = NR5 − LIM3. Percent DV is shown in Supplementary Table S1.

### 2.5. Plan of Analysis

Differences between the energy and nutrient content of MFA products and meat, poultry, fish, and seafood were tested using one-way ANOVA with post hoc Dunnett’s tests. Statistical comparisons were not conducted for categories with excessive missing nutrient values.

## 3. Results

### 3.1. Raw Beef, Pork, and Chicken Meats Compared to MFA Products

The mean energy and nutrient content of raw meat products in the USDA SR28 database are shown in Table 1. The mean energy density was in the 166–183 kcal/100 g range. The mean protein content was 19–20 g/100 g. Total fat content was 11.0 g/100 g for beef, 10.0 g/100 g for chicken, and 9.0 g/100 g for pork. Mean saturated fat content ranged from 2.8 g/100 g (chicken) to 4.4 g/100 g (beef). Beef was highest in iron and zinc and by far the highest in vitamin B12 content. Pork was highest in vitamin B2 (riboflavin), vitamin B6, and selenium. Chicken was highest in vitamin B3 (niacin) and vitamin B5. The sodium content of meat was low (<5% DV). Raw meat did not contain carbohydrates or fiber.

The mean energy density of meat alternatives was 206 kcal/100 g. Mean protein content was 17.4 g/100 g but varied depending on product type (range 0 to 50 g/100 g). The mean total fat content was 8.3 g/100 g, and the mean saturated fat content was 1.5 g/100 g, lower than beef and pork and more similar to chicken. MUFA content was higher than for beef or pork.

Meat alternative products contained carbohydrate (mean 16.8 g/100 g and range 4.3 to 46.4 g/100 g), including mean 4.06 g of total sugar (range 0 to 32 g/100 g). The mean amount of fiber was 3.9 g/100 g (range 0 to 12.9 g/100 g). This is not surprising, as soy, pea, and other textured plant proteins are among the main MFA ingredients. Significantly, MFA products contained high amounts of sodium relative to raw meats. The mean sodium content was 652 mg/100 g (range 54 mg to 2629 mg/100 g), well above that of raw beef, pork, or chicken. However, it should be noted that consumers add salt during the preparation of most meat dishes, while the MFA products often only need to be cooked before consumption. The mean iron was 2.69 mg/100 g for MFA products. Micronutrient values were otherwise available for very few items. There were no values for selenium in MFA products.

**Table 1:** Energy (kcal/100 g) and nutrient content of beef, pork, chicken, and MFA products. Data are means and SD **.

		Beef			Pork			Chicken		MFA Products		
	*n*	Mean	SD	*n*	Mean	SD	*n*	Mean	SD	*n*	Mean	SD
Energy (kcal)	350	183	79	61	166	61	39	171	57	118	205 †	80
Water (g)	350	67.8	7.53	61	70.1	5.8	39	70.6	5.34	5	61.7 †	8.60
Protein (g)	350	20.4	2.59	61	20.1	2.3	39	18.9 †*	2.18	118	17.4 †	7.97
Carbohydrates (g)	350	0.07	0.24	61	0.05	0.16	39	0.03	0.06	118	16.7 †	10.3
Total sugar (g)	350	0.00	0.00	61	0.00	0.00	39	0.00	0.00	118	4.05 †	5.45
Fiber (g)	350	0.00	0.00	61	0.00	0.00	39	0.00	0.00	114	3.86 †	2.07
Total fat (g)	350	11.0	9.86	61	9.01 †	7.74	39	10.0 †	7.09	118	8.12 †	6.60
SAFA (g)	350	4.37	3.90	60	3.00	2.75	39	2.77 †	2.05	115	1.38 †	4.68
MUFA (g)	350	4.95	4.73	61	3.69 †	3.26	39	3.98 †	3.10	32	2.05 †	1.79
PUFA (g)	350	0.46	0.30	61	1.18	1.11	39	2.18 †	1.49	32	2.56 †	2.40
Sodium (mg)	350	59.8	0.64	61	79.1	51.2	39	82.1	28.8	118	660 †	401
Iron (mg)	350	1.97	0.40	61	0.78	0.19	39	0.95	0.35	113	2.69 †	1.80
Vitamin B2 (mg)	319	0.18	0.07	61	0.28	0.08	39	0.17	0.05	11 **	0.16	0.11
Vitamin B3 (mg)	319	4.99	1.30	61	5.85	1.44	39	6.47	1.81	7	4.41	0.89
Vitamin B5 (µg)	287	0.54	0.19	61	0.86	0.26	38	0.98	0.23	8	0.74	0.21
Vitamin B6 (mg)	319	0.49	0.13	61	0.56	0.13	39	0.39	0.12	11	0.22	0.15
Vitamin B12 (µg)	319	2.24	0.92	61	0.61 †	0.19	39	0.47 †	0.24	12	0.95	0.76
Vitamin D (µg)	212	0.09	0.06	51	0.56	0.37	16	0.12	0.10	33	0.00	0.00
Zinc (mg)	319	4.73	1.51	61	2.08	0.52	39	1.44	0.43	13	2.77	1.23
Selenium (µg)	319	20.9	6.89	61	30.7	6.68	39	16.2	3.57	0	--	--
Cholesterol (mg)	350	65.2	8.44	61	63.7	6.48	39	81.0	14.3	0	--	--

**Figure 1:**
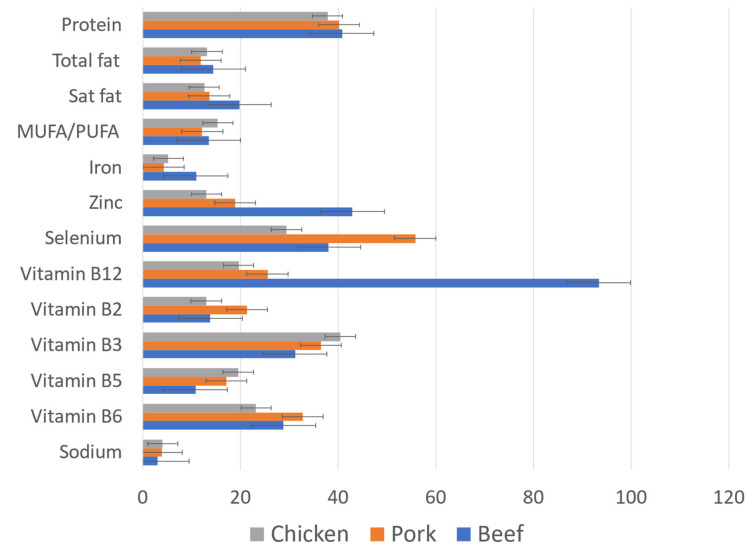
Percent DV of FDA values for raw beef (*n* = 350), pork (*n* = 61), and chicken (*n* = 39).

* † Denotes energy and nutrient means that are not significantly different from MFA products; ** tests were not conducted for categories with excessive missing MFA nutrient values. The selected nutrient content for raw beef, pork, and chicken in the SR28 database is shown in Figure 1. Here, nutrient amounts are expressed as percent US FDA daily values (%DV) per 100 g.

### 3.2. MFA Burgers and Chicken Products

**Figure 2:**
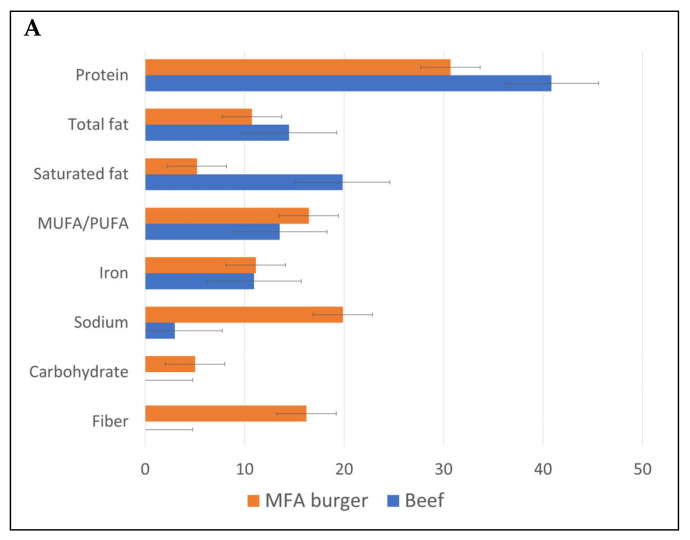

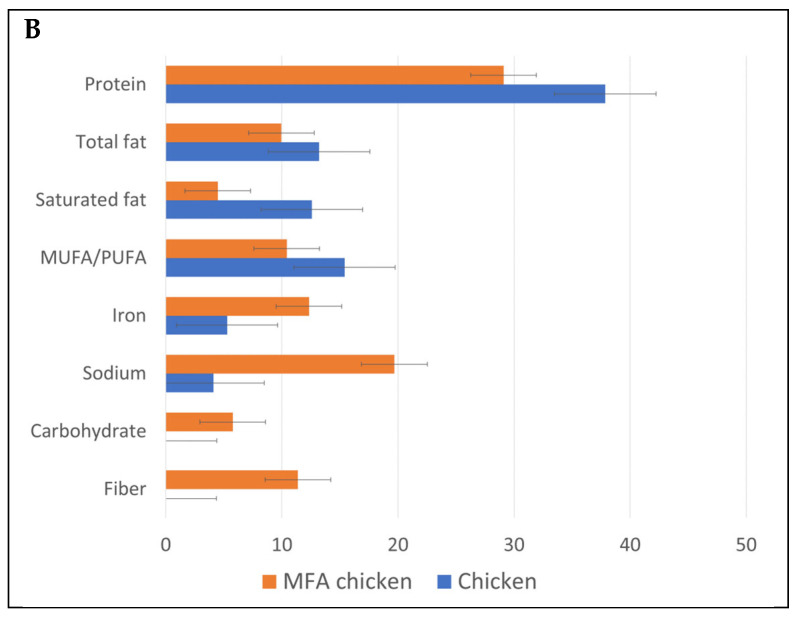
Percentage DV of FDA nutritional reference values per 100 g for raw beef (**A**) and chicken compared to MFA burgers (**B**).

Figure 2A compares the nutrient content of raw beef items (*n* = 350) with MFA meatless burgers (*n* = 26). Figure 2B compares the nutrient content of raw chicken (*n* = 39) with MFA chicken products (*n* = 24). The MFA products were lower in protein, lower in total fat, and much lower in saturated fat. Sodium content was higher compared to beef (mean 20% daily value), and the products contained both carbohydrates and fiber. This product category contained iron (mean > 10% DV per 100 g). Data for zinc and B vitamins were available for a very limited number of items. The rest were either not fortified or did not list the information on the food label.

### 3.3. Nutrient Profiling of Raw Meat and MFA Products by Category

**Figure 3:**
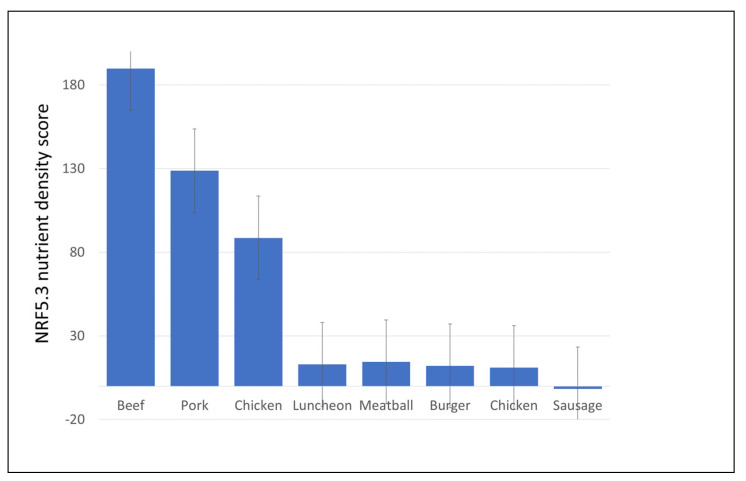
Comparison of raw meats and MFA products on NRF 5.3 nutrient density score.

The nutrient density of raw meat and poultry and of plant-based alternatives was assessed using a variant of the NRF algorithm. The positive NR5 sub score was composed of 5 nutrients to encourage: protein, iron, zinc, selenium, and vitamin B12. The negative LIM sub-score was composed of 3 nutrients to limit: saturated fat, total sugar, and sodium. The values for raw beef, pork and chicken and for the corresponding MFA products are shown in Figure 3. MFA items from the USDA BFPDB database were aggregated into luncheon meats, meatballs, burgers, chicken, and sausage/jerky items. NRF5.3 nutrient density scores were lower for meat alternatives than compared to raw meats and poultry in the USDA database. Means and SD are provided in Supplemental Table S2.

### 3.4. Analysis of BFPD Ingredient Lists for MFA Micronutrient Fortification Patterns

Table 2 shows the main MFA components from the FPDB electronic ingredient list. The most ubiquitous ingredient was added sodium (salt, sea salt), followed by soy isolates and concentrates, gluten and added sugar, methylcellulose, and maltodextrin. Thirty products listed pea as an ingredient, and 15 listed either coconut or coconut oil or palm oil.

About 14 products contained enriched wheat flour, which allowed them to list niacin, ferrous sulfate, thiamine mononitrate, riboflavin, and folic acid in the ingredient list. For six of those products, riboflavin and niacin were also listed in the nutrient composition table; for the remaining eight, they were not. Ferrous sulfate was listed as part of the enriched flour declaration. Another three products listed iron oxide or ferric orthophosphate among ingredients. Since most products did list iron content, it must have come from a protein source, such as mycoprotein, soy protein, or peas.

Seven products listed zinc in the form of zinc oxide. Seven products listed vitamin B12. The listed calcium and magnesium are added for technical rather than nutritional reasons. Based on ingredient lists, the MFA products examined did not seem to be fortified with vitamin B3 (niacin), vitamin B5 or B6, vitamin A, vitamin D, iodine, or selenium.

### 3.5. Nutrient Composition of Fish and Seafood and Plant-Based Alternatives in Frida Database

Comparisons of fish and seafood products (*n* = 68) with very limited (*n* = 12) plant-based alternatives used the Danish Frida food database. Table 3 shows that plant-based alternatives were higher in energy density and contained less protein but more total fat. The plant-based alternatives also contained carbohydrates, fiber, and significantly more sodium (585 mg vs. 309 mg/100 g) compared to fish and seafood. Plant-based alternatives contained less iodine, selenium, zinc, vitamin B12, and vitamin D and did not contain any of the EPA and DHA omega-3 fatty acids.

### 3.6. Nutrient Density of Fish and Seafood and Plant-Based Alternatives in Frida

Table 4 shows the NRF5.3 scores for the fish, seafood, and MFA products in the Frida Database. The positive NR5 sub score was composed of 5 nutrients to encourage: protein, iron, zinc, selenium, and vitamin B12. The negative LIM sub-score was composed of three nutrients to limit: saturated fat, total sugar, and sodium. Similar to raw meat products, NRF5.3 nutrient density scores were lower for MFA items compared to fish and seafood.

### 3.7. Analysis of Ingredient Lists in Mintel for MFA Micronutrient Fortification Patterns

Table 5 shows the items that were found most often on the ingredient lists of fish alternatives (FA). Water, flavors, spices, thickeners, texturants, food acids, and yeast (extracts) were also found often but are not listed below, as the main search was for ingredients that can provide micronutrients or omega-3 polyunsaturated fatty acids. The most common ingredients were salt and sea salt (*n* = 399), sunflower seed oil (*n* = 139), and rapeseed oil (*n* = 133).

Provitamin A as carotene or beta-carotene was added as a colorant to 17 products; vitamin A was not found on the ingredient list. Vitamin B2 (riboflavin) and vitamin B3 (niacin) were added for fortification in 11 and 2 products, respectively. One product contained enriched wheat flour with vitamin B2, and 11 products enriched flour with vitamin B3. Vitamin B5 (*n* = 1), B6 (*n* = 7), B12 (*n* = 33), iodine (*n* = 1), and selenium (*n* = 1) were probably added for fortification purposes in fish alternatives. Iron (*n* = 45) was added for color, fortification, and enriched wheat flour. In three products, zinc was added for nutritional purposes. Calcium and magnesium were added for technical reasons. Calcium was also listed as part of the enriched wheat flour.

## 4. Discussion

Plant-based MFA products, intended to be used regularly in place of meat, poultry, and fish, are becoming part of plant-forward diets at the population level [8]. However, their nutrient quality needs to be addressed using formal methods of nutrient profiling.

Based on the present analyses of a relatively small U.S. database, MFA products were composed of soy isolates and concentrates, pea protein and fiber, gluten, methylcellulose, and maltodextrin. Sodium was a ubiquitous ingredient, present in concentrations of 600 mg/100 g, which is 10 times the level found in raw meat, poultry, or fish. However, it should be noted that processed meat products (i.e., chicken nuggets, burgers, sausages, cold cuts) were excluded. Those products typically contain added salt that is added during processing or as part of cooking at home. A recent study found higher amounts of salt in cold cuts, roasted/cooked red meats, and sausage products compared to their vegan substitutes [31]. Also, seasonings or salt may be added to raw fish during preparation, resulting in higher sodium levels in the final product. Protein was slightly lower in MFA products, but previous studies show that this is not expected to be a problem nutrient for adult vegetarians or vegans [32,33]. The higher fiber and lower saturated fat in MFA products compared to meat are in line with other studies [34,35]. Some fiber was also found in processed meat products, but lower than the vegan substitutes [31].

The sodium difference between the fish, seafood, and MFA products in the Frida Database was smaller but still about twice as much sodium was found in the MFA products. Compared to fish, MFA products in Frida had less protein and more saturated fat. Another study that assessed FA products from Mintel found that protein was lower and saturated fat was comparable to and sometimes lower than in fish [36].

For the MFA products, fortification with iron (14%), zinc (6%), selenium (0%), or vitamin B12 (6%) was rare or non-existent. Also, for FA products, only a limited number were fortified with key nutrients found in fish, such as vitamin B12 (7%), vitamin D (1%), iodine (0.2%), and none with omega-3 EPA and DHA. Previous studies have come to similar conclusions and also showed fortification percentages of less than 10% [36,37]. However, these data can only be viewed as preliminary, given that this market segment is growing rapidly, and new nutrient composition data will become available.

Ideally, MFA products ought to contain the nutrients associated with the original animal-source foods if they are to contribute to healthy and sustainable diets by partially replacing meat and fish [38,39]. Much effort has gone into ensuring that plant-based burgers resemble the appearance, texture, and flavor of the original product, while less effort has been put into matching their nutritional profile [40]. Whether MFA products ought to be called imitations can be addressed through nutrient profiling and resolved through appropriate fortification strategies. No misbranding would occur if the MFA were to have nutrient content that was substantially equivalent to the original product.

The absence of fortification may have regulatory consequences. Regulatory agencies generally frown upon alternative products that are nutritionally too different from the original product. In the US, the Food and Drug Administration (FDA) holds that foods that substitute for and resemble another food but are nutritionally inferior to that food ought to be called “imitations” [39]. That would not be a desirable outcome for the growing industry of plant-based alternative proteins.

However, a lack of regulatory clarity can be a barrier to industry innovation and to consumer acceptance of MFA products. In the US, the federal legal framework for the labeling of alternative proteins is lacking, and two US agencies, the Food and Drug Administration (FDA) and the U.S. Department of Agriculture (USDA), have concurrent and sometimes overlapping jurisdictions. In some cases, the responsibility falls on individual states. Because of regulatory restrictions, the use of meat-like terms to describe non-meat products is permitted in some jurisdictions but not in others [41]. For example, France has banned the use of meat-associated terms to describe plant-based products. A recent FAO/WHO document provides an overview of current country-specific guidelines that are in draft or in place while mentioning that most guidelines are voluntary and do not include guidance on which nutrients to add [42].

The US FDA has not as yet taken a position on MFA products and has not released guidance for the industry on their formulation and labeling. The present analyses of nutrient density may provide a potential first step toward voluntary fortification of MFA products with micronutrients associated with meat, poultry, and fish. Only 6% of meat alternatives were fortified with vitamin B12 and zinc and 14% with iron. Consistent with the present results, a study in Spain found that only 7% of meat alternatives were fortified with vitamin B12 or iron [37]. Another study in Australia [43] found that 20%, 20%, and 16% of meat analogs were fortified with vitamin B12 with vitamin B12, iron, and zinc, respectively. Mintel data show that 5%, 6%, and 7% of meat substitutes launched over the past 5 years in the US were fortified with zinc, iron, and vitamin B12, respectively [26]. The number of fortified FA products was even lower. A recent study that evaluated 149 seafood alternatives from Mintel found that 4% was fortified with micronutrients such as vitamin B12 and iron [36]. A Swiss Market Survey (2024) identified 28 fish alternatives, of which none of them were fortified with iodine, 1 was fortified with vitamin B2, 1 with vitamin D, and 3 with vitamin B12 [44]. Another market survey in the UK identified 11 fish alternatives, and here also, none of the alternatives were fortified with iodine [45]. Other studies [46,47] examined iron and zinc in meat substitutes. The joint FAO-WHO Food Standards Program is evaluating the need for developing guidance for the nutritional composition of MFA products intended to replace traditional foods.

Compared to animal sources of protein, protein-rich legumes (soybeans and pulses), cereals, and seeds are typically low in saturated fats and rich in unsaturated fatty acids, dietary fiber, and multiple phytonutrients [48,49]. Plant-based diets are associated with higher dietary thiamin, vitamin C, E, folate, and magnesium [50]. However, a population shift to plant-based diets also carries the risk—in modeling analyses—of reduced intakes of (bioavailable) iron, zinc, calcium, iodine, and vitamins A, D, and B12 [51,52,53]. As an example, the 2019 EAT-Lancet Commission proposed a largely plant-based global diet, with limited amounts of dairy (250 g/d) and very limited amounts of meat, fish, and eggs (i.e., 29 g/d (62 kcal) of chicken and only 14 g/d (30 kcal) of red meat) [16]. For a 2500 kcal/d diet, the remaining amount of protein (125 g/d and 575 kcal) should be obtained from plant-based sources. Based on subsequent modeling analyses, the EAT-Lancet diet was not only deficient in calcium, iron, zinc, and vitamin B12 [54] but also too costly for the world’s poor [55].

The study had limitations. Plant-based MFA is not well represented in publicly available food composition databases (especially non-fortified, vegan alternatives). The BFPD has the largest number, but it is limited in the number of nutrients it contains. Also, raw meat was compared with plant-based MFA, which is often pre-cooked and requires only limited heat treatment before consumption. Very limited data are available on vitamin B5, and values for multiple other nutrients are missing. Fortification patterns could only be ascertained from analyses of ingredient lists that were sometimes at odds with the listed nutrient composition data. The BFPD and other Food Composition Databases (NEVO, FRIDA and FSANZ) had a very limited number of plant-based fish alternatives. Most fish alternatives are based on the same protein sources as investigated in this study, which implies that they will lack iodine, selenium, vitamin D, and omega-3 EPA and DHA. We found that the main plant-based protein sources of the meat alternatives (delivering the major source of micronutrients) contained some intrinsic minerals; however, the bioavailability of iron and zinc can vary a lot and may be of concern [46,47].

In summary, this study used still-limited nutrient composition data to explore nutrient composition and nutrient profiles of MFA alternatives to traditional meat, poultry, and fish. The analyses highlighted some key nutrients that should be considered when developing new plant-based MFA products. One potential concern is that all plant-based MFA products would be classified as ultra-processed foods under the current NOVA classification scheme [56,57]. Whether plant-based proteins belong in the category of ultra-processed foods is a question that needs to be resolved with some urgency. The provided recommendations and guidelines could be useful for policymakers, producers, and consumers to inform the transition towards more nutritious plant-based options.

## 5. Conclusions

There is a clear need to develop nutrient standards and fortification guidelines for plant-based MFA products, with a focus on protein quality, fortification with nutrients commonly associated with meat, poultry, or fish, and the bioavailability of iron and zinc. Should potentially innovative plant-based products fail such proposed standards, they may be classified by regulatory agencies as imitation meat.

## Figures and Tables

**Table 2:** Major inclusion of ingredients in MFA products based on machine searches of the USDA BFPDB electronic ingredient lists (*n* = 118).

Ingredient	Counts	Ingredient	Counts
Salt, sea salt	118	Vitamin B1 (thiamin)	14
Isolate, concentrate	76	Vitamin B2 (riboflavin)	14
Gluten	69	Vitamin B9 (folic acid)	14
Added sugar	59	Vitamin B12	7
Methylcellulose	26	Calcium	22
Maltodextrin	17	Iron	16
		Magnesium	10
		Zinc	7

**Table 3:** Nutrient composition of fish and seafood (*n* = 68) and plant-based alternatives (MFA) (*n* = 12) in the Danish Frida Food Database (per 100 g). Data are means and SD.

	Fish and Seafood			Fish/Seafood Alternatives		
	N	Mean	SD	N	Mean	SD
Energy (kcal)	68	108	47	12	163 †	44
Water (g)	68	76.5	5.38	12	64.2 †	9.01
Protein (g)	68	17.4	3.24	12	14.8 †	7.09
Carbohydrates (g)	68	0.60	1.13	12	10.4 †	3.53
Fiber (g)	66	0.00	0.00	12	5.51 †	2.18
Total fat (g)	68	3.99	5.60	12	8.20 †	4.53
SAFA (g)	58	0.94	1.46	12	1.57	2.13
MUFA (g)	57	1.69	2.95	12	3.63 †	2.55
PUFA (g)	58	0.97	1.46	12	1.92 †	0.75
Vitamin B2 (mg)	63	0.11	0.09	12	0.20 †	0.13
Vitamin B3 (mg)	61	3.22	2.59	12	0.54 †	0.35
Vitamin B5 (µg)	39	0.68	0.59	12	1.44 †	0.94
Vitamin B6 (mg)	49	0.32	0.34	12	0.05 †	0.02
Vitamin B12 (µg)	51	5.09	5.62	12	0.00 †	0.00
Vitamin D (µg)	64	4.09	6.75	8	0.00 †	0.00
Iron (mg)	63	1.10	1.58	12	3.28 †	4.23
Zinc (mg)	59	2.84	11.0	12	1.87	1.94
Selenium (µg)	63	33.0	17.1	12	9.29 †	8.42
Sodium (mg)	68	309	713	12	585 †	315
Cholesterol (mg)	56	69.2	37.7	12	0.47 †	0.82
Iodine (µg)	62	75.0	110	12	10.9 †	21.6
Linoleic acid (LA), C18:2 n6	48	0.14	0.33	12	1.52 †	0.50
Alpha-linolenic acid (ALA), C18:3 n3	46	0.07	0.14	12	0.40 †	0.32
Eicosapentaenoic acid (EPA), C20:5 n3	37	0.39	0.48	12	0.00 †	0.00
Docosahexaenoic acid (DHA), C22:6 n3	37	0.42	0.49	12	0.00 †	0.00

† denotes significant differences between fish and seafood and MFA at 0.05 level.

**Table 4:** Nutrient density (NR5, LIM3 sub-scores, and total NRF5.3 score) of fish from Frida compared to MFA products. Data are means and SD *.

	N	NR5	SD	LIM3	SD	NRF5.3	SD
Plant-based MFA products	12	81.66 †	32.79	34.20	17.10	47.46 †	38.19
Total fish and seafood	68	163.07	68.00	16.09	20.44	154.70	72.73
Fish lean	21	135.38	55.8	4.6	1.53	134.68	46.05
Fish medium oily	8	141.31	65.47	6.70	2.25	134.61	66.49
Fish oily	12	152.60	60.08	24.76	33.14	137.89	78.03
Mollusks	4	243.28	102.94	23.40	13.90	256.40	52.95
Shellfish	11	205.58	80.60	32.71	16.14	188.89	85.38

* Tests compare oktotal fish and seafood and MFA products. † Denotes significance at 0.05 level.

**Table 5:** Major ingredients are listed on the ingredient list of fish alternatives (FA) based on ingredient lists in Mintel GNPD (*n* = 503).

Ingredient	Counts	Ingredient	Counts
Salt, sea salt	399	Vitamin A	0
Sunflower seed oil	139	Provitamin A (carotenes)	17
Rapeseed oil	133	Vitamin B2 (riboflavin)	13
Methylcellulose	120	Vitamin B3 (niacin)	13
White sugar	85	Vitamin B5	1
Pea protein	70	Vitamin B6	7
Wheat gluten	64	Vitamin B12	33
Wheat protein	52	Vitamin D	7
Soy protein concentrates	48	Iron	45
Linseed oil	47	Zinc	3
Algae	46	Calcium	54
Soybean, soybean proteins	99	Magnesium	18
Wheat, corn, and rice flour	209	Selenium	1
Vegetable oils	41	Iodine	1

## Data Availability

USDA nutrient composition data are available on FoodData Central. Frida food composition data are available on frida.fooddata.dk.

## Supplementary Materials

The following supporting information can be downloaded at https://www.mdpi.com/article/10.3390/nu16162725/s1, Table S1: Reference Values per day for adults based on a 2000 kcal diet for labeling purposes [58,59,60,61,62]. Table S2: Nutrient density (NR5, LIM3 sub-scores and total NRF5.3 score) of raw beef, pork, and chicken compared to MFA products by category.
