# Supplementary material for: Comparing Nutrient Profiles of Meat and Fish with Plant-Based Alternatives: Analysis of Nutrients, Ingredients, and Fortification Patterns
Source: Nutrients. 2024 Aug 16;16(16):2725. doi: 10.3390/nu16162725 (PMC11357199; doi:10.3390/nu16162725)
Supplement: Supplementary file 1 [file nutrients-16-02725-s001.zip › nutrients-3148866-supplementary.pdf]

Comparing Nutrient Profiles of Meat and Fish with Plant-Based Alternatives: Analysis of Nutrients, Ingredients, and Fortification Patterns

Adam Drewnowski [1,*], Maaike J. Bruins [2] and Julia J. F. Besselink [2]

Supplemental Tables

Supplemental Table S1. Reference Values per day for adults based on a 2000 kcal diet for labelling purposes from the FDA (Daily Value, DV) [58], the Recommended Daily Allowances (RDA) as set by EU for labelling purposes for micronutrients and macronutrients [59,60] and ALA, LA, EPA plus DHA as set by EFSA [61] and mono- and polyunsaturated fat from the Academy of Nutrition and Dietetics (AND) [62].

	FDA (DV)	EU (RDA)	EFSA	AND
Energy (kcal)	2000	2000	ARs only	
Protein (g)	50	50	ARs only	
Carbohydrates	275 g	260 g	45-60 E%	
Sugar (g)		90		
Added sugar (g)	50			
Dietary fiber (g)	28		25 (AI)	
Total fat	78 g	70 g	20-35 E%	
Cholesterol (mg)	300			
Saturated fat (g)	20	20	ALAP	
Mono- and polyunsaturated Fat (E%)				19-31%
Alpha-linolenic acid (ALA) (E%)			0.5 (AI)	
Linoleic acid (LA) (E%)			4 (AI)	
Docosahexaenoic acid (DHA) plus Eicosapentaenoic acid (EPA) (mg)			250 (AI)	
Vitamin B2 (riboflavin) (mg)	1.3	1.4		
Vitamin B3 (niacin) (mg NE)	16	16		
Vitamin B5 (pantothenic acid) (mg)	5	5		
Vitamin B6 (pyridoxine) (mg)	1.7	1.4		
Vitamin B12 (µg)	2.4	2.5		
Vitamin D (µg)	20	5		
Iodine (µg)	150	150		
Iron (mg)	18	14		
Zinc (mg)	11	10		
Selenium (µg)	55	55		
Sodium (mg)	<2300	2400	<2000 (SAI)	

AR: Average Requirement; AI: Adequate Intake; ALAP: As Low As Possible; E%: Energy percentage, SAI: Safe and Adequate Intake

Supplemental Table S2. Nutrient density (NR5, LIM3 sub-scores and total NRF5.3 score) of raw beef, pork, and chicken compared to MFA products by category.

	N	NR5	SD	LIM	SD	NRF5.3	SD
Beef	350	210.81b	71.80	21.38b	16.14	188.91b	80.18
Pork	61	144.83b	13.75	16.65b	12.43	128.48b	22.10
Chicken	39	105.33b	12.25	15.47b	8.65	88.60b	17.24
Total MFA	113	49.00a	21.72	40.33a	22.79	8.91a	18.10
Luncheon meat	10	61.44	20.72	48.69	11.10	17.98	21.56
Meatball	23	43.25	13.82	29.46	7.69	14.52	11.48
Burger	24	40.82	15.39	28.71	8.78	12.11	17.50
Chicken	25	42.43	12.67	31.36	14.28	11.05	18.49
Sausage/jerky	34	58.84	29.08	61.20	29.54	-1.57	17.35
